# Surgery for complete atrioventricular septal defect: Is a uniform strategy applicable?

**DOI:** 10.4103/0974-2069.52811

**Published:** 2009

**Authors:** Sachin Talwar, Shiv Kumar Choudhary, Balram Airan

**Affiliations:** Department of Cardiothoracic and Vascular Surgery, All India Institute of Medical Sciences, New Delhi, India

This issue of the Annals of Pediatric Cardiology contains two very different approaches for repair of complete atrioventricular septal defect (CAVSD). Although Dr. Backer's group from Chicago[1] has made a case for the “modified single-patch technique” which is also popularly known as the “Australian technique”, Dr. Mosca from New York[2] has presented his views on the traditional “two-patch technique” for repair of CAVSD. In this backdrop, it is only appropriate that we discuss the other important approach, the “One patch technique,” which has also been popularized by many groups.[3,4] At the same time, it may be worthwhile to review recent publications on the anatomy of the CAVSD and its relevance to optimal surgical strategy.

Adachi *et al.*[5] from the Royal Brompton Hospital, London, United Kingdom, recently examined 43 heart specimens: Thirty-one with CAVSD and 12 with partial atrioventricular septal defect (PAVSD). In these hearts, they assessed the configuration of the superior bridging leaflet and its subvalvar apparatus (SVA), relative to the ventricular crest, according to the traditional Rastelli classification.[6] All these hearts had concordant atrioventricular and ventriculoarterial connections in the setting of the usual atrial arrangement; those with associated anomalies were excluded. The authors measured the size of the ventricular component of the septal deficiency (scoop) and the diameter of the left ventricular outflow tract (LVOT) relative to the width of the atrioventricular (AV) junction. The ventricular component was also studied, to determine whether it extended beyond the level of the anterosuperior end of the AV junction. The depth of the scoop was defined as the distance from the ventricular crest to the AV valve.

Nineteen patients had Rastelli Type A, two had Rastelli type B, and seven had Rastelli type C form of CAVSD. In three patients the Rastelli subtype could not be defined. Out of the 31 specimens with CAVSD, 16 (52%) had anterosuperior extension of a ventricular component of the septal deficiency; hearts with PAVSD did not exhibit this pattern. Six out of the seven hearts with Rastelli type C defect were associated with the anterosuperior extension of the scoop, while only seven out of the 19 hearts with Rastelli type A anatomy exhibited this extension. Hearts with CAVSD and anterosuperior extension had a significantly deeper scoop than CAVSD without the extension; also the scoop tended to be deeper in the CAVSD with anterosuperior extension than in PAVSD. There were no differences between CAVSD without the extension and PAVSD. These findings indicate that the deepest part of the scoop was principally beneath the superior bridging leaflet in the CAVSD with the anterosuperior extension, whereas, the deepest part of the scoop was approximately centrally located in the other groups. Therefore, the deepest part of the scoop was anterosuperiorly skewed in hearts with the extension and not symmetric as compared to the other groups.

Hearts with CAVSD and anterosuperior extension had a significantly narrower LVOT than the complete form without the extension and the partial form. Therefore, the authors concluded that hearts with CAVSD with a deeper scoop are more likely to have a narrower LVOT. However, when the LVOT diameters were compared between hearts with CAVSD having a deeper scoop (defined as depth of 60% or greater) and those having a shallower scoop (depth of less than 60%), the LVOT diameters did not differ significantly suggesting that that the depth of the scoop was not always a reliable marker for a narrow LVOT.

Viewed in the context of the findings of this study, it may not be prudent to make a generalization as to the universal applicability of the modified single-patch technique, despite its claimed advantages of simplicity, reproducibility, shorter operating time, avoidance of division of the valve leaflets, and avoidance of the insertion of rigid prosthetic patch material in the LVOT, which is presumably thought to prevent turbulence and fibrosis, particularly if the LVOT is intrinsically narrow. However, the results obtained from this study seem to indicate that from a morphological point of view, the LVOT diameter would inevitably become narrower with the simplified technique, because this technique essentially converts the CAVSD into a PAVSD, in which the LVOT is inherently narrower.

The authors of this article have also suggested guidelines for determining the suitability or unsuitability of the modified single-patch technique. Hearts with double outlet right ventricle type of anatomy are unsuitable for this technique because the only outlet for the left ventricle is the space between the bridging leaflets and the ventricular crest, and the use of the simplified technique in such situations can reduce this space and precipitate LVOT obstruction. In hearts with ventriculoarterial concordance, the modified single-patch technique has traditionally not been recommended if the depth of the scoop is more than 10 mm, as there may be distortion of leaflets and excessive tension may be required to bring the leaflets to the septum in these patients.[7] Adachi *et al.*[5] have also shown that because the anterosuperior border of the defect constitutes a substantial portion of the LVOT, the anterosuperior extension of the scoop is more important than its depth as it directly affects the size of the LVOT; a deep scoop does not necessarily indicate a narrow LVOT. At the same time, a relatively shallow scoop does not always exclude the risk of LVOT obstruction. The depth of the scoop and its extension must therefore be carefully assessed by the surgeon in the operating room, as echocardiography may fail to detect this finding. Moreover, patients with Rastelli Type C anatomy are extremely likely to have this extension and may probably develop LVOT obstruction. Such patients would probably be better candidates for the traditional two-patch repair or the “classical one-patch procedure”.

The major drawback of this study is, however, the lack of clinical data to support these morphological findings. Here we may also like to add that reports by Nunn[8] and Backer[9] have demonstrated the uniform applicability of this technique (although they have not specifically addressed these morphological subtypes) with a median follow-up of just around seven years. Although the results are encouraging so far, it will take more follow-up and careful assessment of the individual subtypes to decide how they fare in the long term.

In this backdrop, we reviewed a recent article by Draulescu *et al.*[10] These authors evaluated their results with the Rastelli one-patch repair for CAVSD. Between 1984 and 2005, the records of 107 patients with CAVSD, undergoing a Rastelli one-patch procedure, at the Children's Hospital, La Timone, Marseille, France were retrospectively reviewed. Preoperatively, 67 patients had absent or trivial left-sided AV valve regurgitation (LAVVR), 28 had minimal, 10 had moderate, and two had severe LAVVR.

Surgical procedure included division of the common anterior leaflet in type C and extension of the native division in type A, as well as, division of the common posterior leaflet. A single glutaryldehyde treated pericardial patch was sutured on the right side of the ventricular septum and the left-and right-sided AV valve components were attached to the pericardial patch. The cleft in the left AV valve was not closed in 70 patients, whereas, it was closed partially in 29 patients, and completely in eight patients. The authors[8] preferred to leave the cleft open if there was a potential parachute left AV valve, if the valvular tissue was thin and fragile, and if there was satisfactory apposition, closure, and competence of the valve, tested after injecting saline into the left ventricular cavity. They closed it completely if the free edges of the cleft were thickened or dysplastic or if there was severe preoperative LAVVR. They closed the cleft partially for intermediate situations. Following this, the atrial septal defect was closed with the same patch. A right ventricular outflow patch was placed in 11 patients with pulmonary obstruction, and coarctation repair, with end-to-end resection anastomosis was performed in three patients.

The in-hospital mortality for the entire group was 13%; in the last 10 years significant advances have been made in the intensive care unit management of patients with postoperative pulmonary hypertensive crisis and the early mortality dropped to less than 4%. All patients were in normal sinus rhythm. Five patients required early reoperation: Three for severe LAVVR and two for significant residual shunt. The mean follow-up was 9.5 ± 6.9 years (range of 2 to 23 years). There were three late deaths due to unrelated causes. The actuarial survival at 10, 15, and 20 years was constant at 84.1%. Actuarial freedom from reoperation for LAVVR was 96.7% at 5 years, 94.6% at 10 years, and 92% at 15 and 20 years. Nine patients required reoperation: Four for development of subaortic stenosis and five for LAVVR. Surprisingly the partial or complete closure of the cleft did not affect the degree of LAVVR and rate of reoperation for LAVVR. However, this is an area of controversy, as many groups have advocated routine closure of the cleft in all patients.[11,12]

The excellent results obtained by this group over a 20- year period[10] and by many others[4], do indicate that the “one-patch technique” is another suitable option for patients with CAVSD. We, however, do believe that late outcomes following repair of CAVSD are multifactorial and it is advantageous for a surgeon to be familiar with all the three options, for managing these technically challenging patients. A good understanding of the anatomy followed by careful selection of the appropriate technique will help to achieve the most stable long-term results.
